# Emergence and melting of active vortex crystals

**DOI:** 10.1038/s41467-021-25545-z

**Published:** 2021-09-24

**Authors:** Martin James, Dominik Anton Suchla, Jörn Dunkel, Michael Wilczek

**Affiliations:** 1 Max Planck Institute for Dynamics and Self-Organization (MPI DS), Göttingen, Germany; 2 Faculty of Physics, University of Göttingen, Göttingen, Germany; 3 Department of Mathematics, Massachusetts Institute of Technology, Cambridge, MA USA

**Keywords:** Physics, Biological physics, Fluid dynamics, Statistical physics, thermodynamics and nonlinear dynamics

## Abstract

Melting of two-dimensional (2D) equilibrium crystals is a complex phenomenon characterized by the sequential loss of positional and orientational order. In contrast to passive systems, active crystals can self-assemble and melt into an active fluid by virtue of their intrinsic motility and inherent non-equilibrium stresses. Currently, the non-equilibrium physics of active crystallization and melting processes is not well understood. Here, we establish the emergence and investigate the melting of self-organized vortex crystals in 2D active fluids using a generalized Toner-Tu theory. Performing extensive hydrodynamic simulations, we find rich transition scenarios. On small domains, we identify a hysteretic transition as well as a transition featuring temporal coexistence of active vortex lattices and active turbulence, both of which can be controlled by self-propulsion and active stresses. On large domains, an active vortex crystal with solid order forms within the parameter range corresponding to active vortex lattices. The melting of this crystal proceeds through an intermediate hexatic phase. Generally, these results highlight the differences and similarities between crystalline phases in active fluids and their equilibrium counterparts.

## Abstract (web summary)

Whereas transitions from solid- to fluid-like states in systems of active particles have received much attention, the characterization of phase transitions in active fluids with self-organized vortices so far has remained elusive. James et al. take us on a numerical tour de force from active turbulence to active vortex crystals.

## Introduction

Melting of 2D crystal structures has played a pivotal role for our understanding of order-disorder transitions in equilibrium systems^1^. It remains an open question, however, to which extent such transitions generalize to crystallization and melting phenomena in active systems. In contrast to equilibrium crystals, active non-equilibrium crystals can both self-assemble^2,3^ and melt into an active fluid^4–6^, owing to the intrinsic motility of their microscopic constituents. The spontaneous emergence and destruction of crystal-like order can be observed in a wide variety of natural and artificial systems^2,7–10^. Striking examples range from suspensions of active colloids^2,11,12^, bacteria^7^ and sperm cells^9^ to biological tissues^8^ that can solidify and fluidize during embryonic development^13,14^. Yet, despite recent experimental advances^7–9,13,15,16^ and important theoretical progress^6,13,14,17–26^, many key aspects of active melting processes remain poorly understood. This may not come as a surprise given that it took several decades to decipher the complex melting scenarios of even the most basic 2D equilibrium crystal structures^1^.

In thermal equilibrium, 2D crystalline solids exhibit quasi-long-range positional and long-range bond-orientational order at low temperatures. As the temperature is increased beyond a critical value, such crystals lose their order and transition to a liquid state^27–30^. Over the last decades, several theories on the type and nature of phase transitions in 2D equilibrium systems have been proposed^1^. The seminal work by Kosterlitz, Thouless, Halperin, Nelson, and Young (KTHNY)^31^ predicted a two-step continuous melting transition that proceeds through an intermediate hexatic phase characterized by quasi-long-range orientational order and short-range positional order. However, recent advances have shown that the liquid-hexatic transitions in equilibrium hard-disk systems can be discontinuous^32^. Vortex lattices in superconductors have also been demonstrated to undergo a discontinuous transition^33^ as well as a dynamic melting^34^. Hexatic phases have been observed in experiments on colloidal systems^35^ and superconducting lattices^36^ as well as in numerical simulations of repulsive disks^37^.

The complex melting dynamics of 2D equilibrium crystals, combined with recent advances in the control of synthetic^2,11,12,38^ and natural^7^ active matter, have stimulated an intense interest in phase transitions in far-from-equilibrium systems^6,39–43^. Recent experimental and numerical studies of particulate active matter provide evidence for an equally if not even more complex phenomenology than in passive systems. For example, Monte Carlo simulations for active particles with inverse-power-law repulsion^4^ showed an intermediate hexatic phase. Agent-based simulations^5^ and active Brownian particle simulations^6^ further suggest that active crystal structures can melt into a hexatic phase without the KTHNY-typical unbinding of topological defect pairs.

Active fluids constitute another important class of non-equilibrium systems that exhibit intriguing transitions from active turbulence^44–48^ to highly ordered vortex lattices. Vortex lattices have been observed in dense suspensions of swimming sperm cells^9^ or microtubule^49^, and have been predicted to form spontaneously by a wide range of generic active fluid models^45,45,46,48,50–55^. However, so far it has not been established whether such active systems can exhibit crystalline order at macroscopic scales, and if so, how active vortex crystals (AVCs) melt.

The systematic study of AVC formation and melting has remained a challenge owing to the large system sizes and simulation times required. Overcoming previous limitations through large-scale direct active fluid simulations, we report here a detailed computational investigation of AVC emergence and melting in an experimentally validated generalized Toner-Tu model^44,56^. By evaluating the corresponding order parameters, we establish conclusively that the active vortex lattices in two dimensions can indeed self-organize into a solid phase with long-range orientational order. Our analysis further reveals a rich spectrum of dynamics including hysteresis between different dynamical states and a solid-hexatic-liquid transition as the system approaches the thermodynamic limit. The hexatic phase appears remarkably robust, persisting over a range of activity parameters. We also find that the emergence of AVCs shows intriguing transient features as a result of the self-organization of AVCs through a turbulent transient, followed by the slow coarsening dynamics of large active vortex domains of opposite polarity.

## Results

### Active fluid model

Our starting point is a generalization of the incompressible Toner-Tu equations^57–59^ for the active fluid velocity field **u** and pressure field *p*^44,45,56^, which take the nondimensionalized form:

1 $${\partial }_{t}{{{{{{{\bf{u}}}}}}}}+\lambda {{{{{{{\bf{u}}}}}}}}\cdot \nabla {{{{{{{\bf{u}}}}}}}}	= \, -\nabla p-{(1+{{\Delta }})}^{2}{{{{{{{\bf{u}}}}}}}}-(\alpha +\beta | {{{{{{{\bf{u}}}}}}}}{| }^{2}){{{{{{{\bf{u}}}}}}}},\\ \nabla \cdot {{{{{{{\bf{u}}}}}}}}	= \, 0,$$

where *λ* is an active advection parameter which incorporates the effects of active nematic stresses^45,60,61^, *α* < 0 is the activity parameter and *β*, which can be scaled out, is set to 0.01 for all simulations. Active flows described by (1) are self-driven through a linear instability induced by the Swift-Hohenberg operator (1 + Δ)^2^, which favors periodic flow patterns of wave length 2*π*^62,63^. Equation (1) can be derived from a generic agent-based model^60,61^ that accounts for the particles’ self-propulsion, their hydrodynamic interactions, and their steric interactions. It has been shown^44^ that this minimal continuum theory quantitatively captures essential statistical properties of dense bacterial suspensions in quasi-2D microfluidic chambers. Here, we apply (1) to study AVC dynamics by performing large-scale simulations on a doubly periodic domain of size *L* × *L* with *L* ranging up to 1000*π* using a pseudo-spectral method for spatial discretization and a fourth-order Runge-Kutta scheme for time stepping (Methods).

### Dynamical states of the active fluid model

**Fig. 1:**
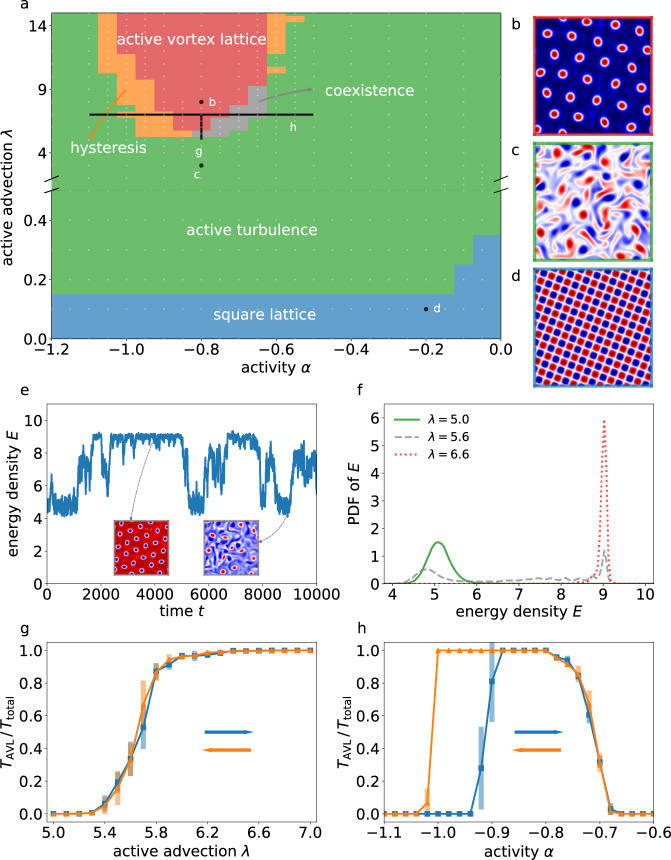
Dynamical states and their transitions in the active fluid model (domain size *L* = 20*π*). (**a**) Dynamical states as a function of activity and active advection, obtained from close to 1000 simulations (Methods). Red, green, and blue regions correspond to (**b**) active vortex lattice (AVL), (**c**) active turbulence (AT) and (**d**) square lattice states, respectively. The gray and orange regions are the marginal stability regions between the AT and the AVL states. The white dots show the parameter configurations used to obtain the phase diagram (see also Fig. 6). (**e**) A typical energy density time series for a simulation in the marginal stability region (*α* = −0.8, *λ* = 5.6) illustrates the intermittent melting and organization of the AVL. The insets show representative snapshots of the vorticity field. (**f**) Probability density functions of the energy density for values of *λ* = 5 (green), 5.6 (gray) and 6.6 (red). (**g**) AVL-AT transition as a function of active advection (*α* = −0.8, vertical black line in (**a**)) and (**h**) transition along the activity axis (*λ* = 7, horizontal black line in (**a**)). The blue and orange curves correspond to increasing and decreasing values, respectively, of *α* and *λ*. The error bars signify the standard deviations calculated from five independent datasets (Methods).

We performed ~ 1000 simulations on smaller domains of size *L* = 20*π* to map out the dynamical states and their transitions in the (*α*, *λ*) space shown in Fig. 1a. These parameter scans revealed three distinct states: The active vortex lattice (AVL) state (Fig. 1b) forms for *α* ≈ −0.8 and sufficiently large values of the active advection parameter *λ*, corresponding to strong extensile stresses^45^ (red-colored domain in Fig. 1a). In this state, vortices of the same spontaneously chosen handedness self-organize in a triangular lattice, phenomenologically similar to those observed in dense sperm suspensions^9^. Strikingly, this spontaneous symmetry breaking occurs after an initial turbulent transient [Supplementary Video (1)]. The wavenumber of the lattice thereby is smaller than that of the linearly most unstable mode, which can be rationalized by an inverse energy transfer from smaller to larger scales^48^. Note that AVL refers to an ordered vortex array. Only if additionally long-range orientational order can be established, we call it an active vortex crystal (AVC). The AVL state is surrounded by an extended active turbulence (AT) state (green domain in Fig. 1a), in which transient vortices of either handedness coexist in the fluid (Fig. 1c). Finally, for low active advection *λ* ≪ 1, corresponding to contractile stresses^45^, the system settles into a stationary square flow-lattice state (Fig. 1d), which can be explained with classical pattern formation theory^48^. We focus in the following on the transitions between the AVL and AT states, corresponding to the regions separating the red and green domains in Fig. 1a.

Interestingly, our simulations reveal two distinct AVL-AT transition scenarios, characterized by coexistence and hysteresis, respectively. To demonstrate the characteristics of AVL-AT coexistence (gray in Fig. 1a), we keep the activity parameter *α* = −0.8 fixed and decrease the active advection parameter *λ* (vertical scan). This transition is characterized by an intermittent switching between active turbulence and AVLs [Supplementary Video (2)]. The energy density time series of a corresponding simulation is shown in Fig. 1e. In the AVL state, the energy density is high due to the close packing of vortices, and the fluctuations are low. In the AT state, the energy density is lower and fluctuations are larger. The energy-density probability density functions (PDFs) for three representative intermediate values of *λ* along the vertical scan quantify the relative abundance of each state (Fig. 1f). This temporal, highly dynamic phase coexistence is confirmed by measuring the fraction of time in the AVL state, which is shown for the vertical scan in Fig. 1g.

To illustrate the hysteretic transition (orange in Fig. 1a), we keep the active advection fixed at value *λ* = 7 and change the activity parameter *α* (horizontal scan). In the transition region for large negative activity parameters, the AVL will not emerge from random initial conditions, but the AVL itself is a stable solution. This is illustrated by the AVL time fraction for the horizontal scan shown in Fig. 1h, which clearly exhibits a hysteresis loop. As the activity parameter is further increased, a second transition through the coexistence region without hysteresis is observed. Closer to the boundary of the active turbulence region, the vortex lattices start showing a liquid-like arrangement of vortices rather than solid-like or hexatic. We stress that the system size considered so far is not sufficient to clearly establish phases and transitions in the thermodynamic sense. For a systematic characterization in terms of such an analysis, we present results from significantly larger domains in the following sections.

### Solid, liquid and hexatic phases

We perform simulations that are more than three orders of magnitude larger than for the diagram mapping out the dynamical states (*L* = 1000*π*). We choose three exemplary parameter sets to see how the transition from an AVL to active turbulence translates to larger domains. Two of the parameter sets (*λ* = 15, *α* = −0.9 and *λ* = 7, *α* = −0.75) correspond to the AVL regime on small domains, whereas the third one (*λ* = 7, *α* = −0.7) falls into the coexistence region.

**Fig. 2:**
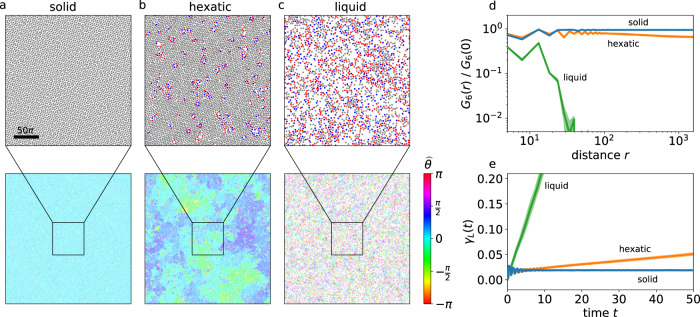
Characterization of different phases on a *L* = 1000*π* domain. Top row: Vortex cores (gray) and the locations of the 5-fold (red) and 7-fold (blue) defects in the (**a**) solid (*α* = − 0.9, *λ* = 15), (**b**) hexatic (*α* = − 0.75, *λ* = 7) and (**c**) liquid (*α* = − 0.7, *λ* = 7) phases. The scale bar denotes a length of 50*π*. Bottom row: The local orientational order, visualized by the deviation $\hat{\theta \,}$ from the mean orientation angle (Methods), becomes increasingly short-ranged from the solid, to the hexatic, and the liquid phases. (**d**) The orientational correlation function *G*_6_(*r*) remains constant in the solid phase, decays approximately algebraic in the hexatic phase and faster than algebraic in the liquid phase. (**e**) Dynamic Lindemann parameter *γ*_*L*_(*t*) as a function of time. *γ*_*L*_(*t*) remains approximately constant for the solid phase, whereas it diverges for both the hexatic and liquid phases. The envelope around each line signifies the standard deviation, calculated from six snapshots for (**d**) and six ensembles of vortex core trajectories for (**e**) from the same simulation at different times.

To characterize the different phases, we construct a Voronoi partition to identify 5-fold and 7-fold defects (Methods). Deep inside the AVL regime, we observe a well-ordered structure with no dislocations (Fig. 2a: *λ* = 15, *α* = −0.9). Closer to the active turbulence region, the AVL is less ordered, and we observe the unbinding of dislocation pairs, as well as a few free disclinations (Fig. 2b: *λ* = 7, *α* = −0.75). As we move closer to the active turbulence region clusters of defects emerge (Fig. 2c: *λ* = 7, *α* = −0.7).

To characterize these different phases, we evaluate the orientational order. As a first step, we calculate the orientational order ${\psi }_{i}={\sum }_{j}\exp (6{{{{{{{\rm{i}}}}}}}}{\theta }_{ij})/N(i)$ for each lattice site *i*. Here, *θ*_*ij*_ is the angle between the line connecting the neighbors *i* and *j* and an arbitrary axis, and *N*(*i*) is the number of neighbors. The orientational order allows to calculate a local deviation from the mean orientation angle (Methods). In the bottom row of Fig. 2a–c the local orientation is shown for the same choice of parameters as in the top row, indicating that the local orientational order is becoming increasingly short-ranged from a–c. This can be quantified in terms of the orientational correlation *G*_6_(*r*)^1^, defined by

2 $${G}_{6}(r)=\frac{\left\langle {\psi }_{i}^{* }{\psi }_{j}\delta (r-{r}_{ij})\right\rangle }{\left\langle \delta (r-{r}_{ij})\right\rangle }$$

where *r*_*ij*_ is the distance between vortex cores *i* and *j*, and the average is taken over all lattice sites *i* and *j*. Figure 2d shows the orientational correlation function for the different parameters. Well inside the AVL regime, we observe long-range orientational order, suggesting solid order. Closer to the transition region, *G*_6_(*r*) shows quasi-long-range order characterized by an algebraic decay, which is indicative of a hexatic phase. For the third set of parameters, corresponding to the coexistence region on small domains, the orientational correlation function decays faster than algebraic, indicating a liquid phase.

As an additional characterization, we use the dynamic Lindemann parameter, which is defined as the relative displacement of neighboring vortex cores^35,64,65^:

3 $${\gamma }_{L}(t)=\frac{\left\langle {({{\Delta }}{{{{{{{{\bf{x}}}}}}}}}_{i}(t)-{{\Delta }}{{{{{{{{\bf{x}}}}}}}}}_{i+1}(t))}^{2}\right\rangle }{2{a}^{2}}.$$

Here, Δ**x**_*i*_(*t*) = **x**_*i*_(*t*) − **x**_*i*_(0) is the temporal displacement of a vortex core position **x**_*i*_(*t*) from its initial position **x**_*i*_(0), *i* and *i* + 1 denote neighbors, and *a* is the lattice spacing. For a solid, *γ*_*L*_(*t*) remains bounded whereas for both hexatic and liquid phases, it diverges with time. Figure 2e shows that the dynamic Lindemann parameter for our system remains approximately constant well inside the AVL regime, further supporting the identification of a solid phase. Close to the transition region, *γ*_*L*_(*t*) diverges, indicating either a hexatic or a liquid phase.

Taken together, based on the indications for solid order deep in the AVL regime, we conclude that part of the AVL regime corresponds to an active vortex crystal (AVC) regime. Closer to the transition region, we find evidence for a hexatic phase within the AVL regime. Finally, within the coexistence region on small domains, we find a liquid phase on large domains. Overall, this shows that this solid-to-liquid transition proceeds through an intermediate hexatic phase.

### Phase transitions

**Fig. 3:**
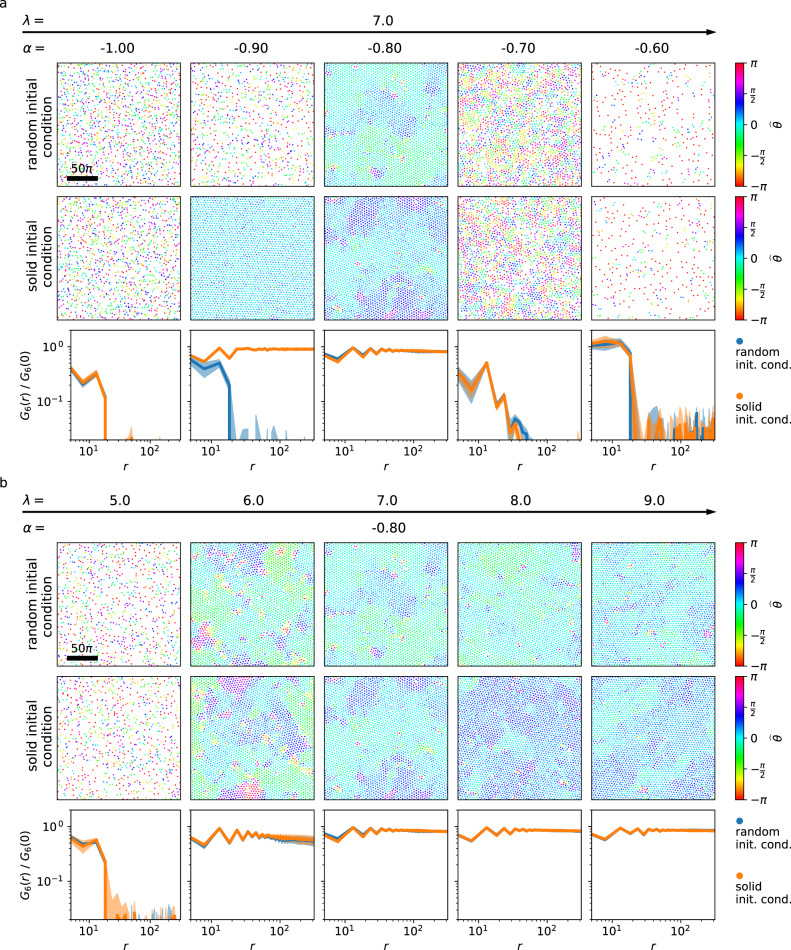
Phase transitions on a *L* = 200π domain. Results obtained from random (top row in (**a**) and (**b**)) and solid (middle row in (**a**) and (**b**)) initial conditions. (**a**) For *λ* = 7 and increasing *α*, the orientational correlation functions (bottom row) suggest a hysteretic transition from the liquid to the hexatic phase, followed by a non-hysteretic transition from the hexatic phase to the liquid phase. In the latter transition region, ordered vortex clusters coexist with an ambient liquid phase. (**b**) For increasing *λ* and *α* = −0.8, we observe a transition from the liquid phase, over the hexatic phase, to the solid phase. The standard deviation of *G*_6_, calculated from three snapshots of the same simulation at different times, is plotted as an envelope.

To confirm that the transitions between the various dynamical states mapped out in Fig. 1a translate to the transitions between phases on larger domains, we consider the orientational correlation function along two cuts through parameter space (*λ* = 7, *α* ∈ [−1.0, −0.6], and *λ* ∈ [5.0, 9.0], *α* = −0.8) on *L* = 200*π* domains. For each set of parameters, we run two simulations, one with random initial conditions and one starting from the solid phase. Figure 3a shows the cut for fixed *λ*. For *α* = −1.0, we find a liquid phase, irrespective of the initial conditions. For *α* = −0.9, we observe hysteresis, i.e. either a liquid or a solid phase, depending on the initial condition. This matches the hysteresis region found on small domains. For *α* = −0.8, we find a hexatic phase, irrespective of the initial condition, which corresponds to the AVL state found on small domains. For *α* = −0.7, the corresponding snapshots in Fig. 3a illustrate the spatial coexistence between well-ordered and disordered regions, reminiscent of local hexatic and liquid order, respectively, which is also reflected in the decay of the the orientational correlation function (Fig. 3a, bottom). This corresponds to the temporal coexistence region found on small domains. Finally, for *α* = −0.6, we observe a liquid phase irrespective of the initial condition, matching the active turbulence state found on small domains. The vertical cut varying *λ* for *α* = −0.8 is shown in Fig. 3b. Here we observe a transition from the liquid phase (*λ* = 5) via a hexatic phase (starting at *λ* ≈ 6) to the solid phase starting around *λ* ≈ 9. As expected from the result on small domains, no hysteresis was observed for this transition. Overall, these results show that the transitions between the dynamical states on small domains correspond to transitions between liquid, hexatic and solid phases on large domains.

### Emergence of AVLs as function of the domain size

**Fig. 4:**
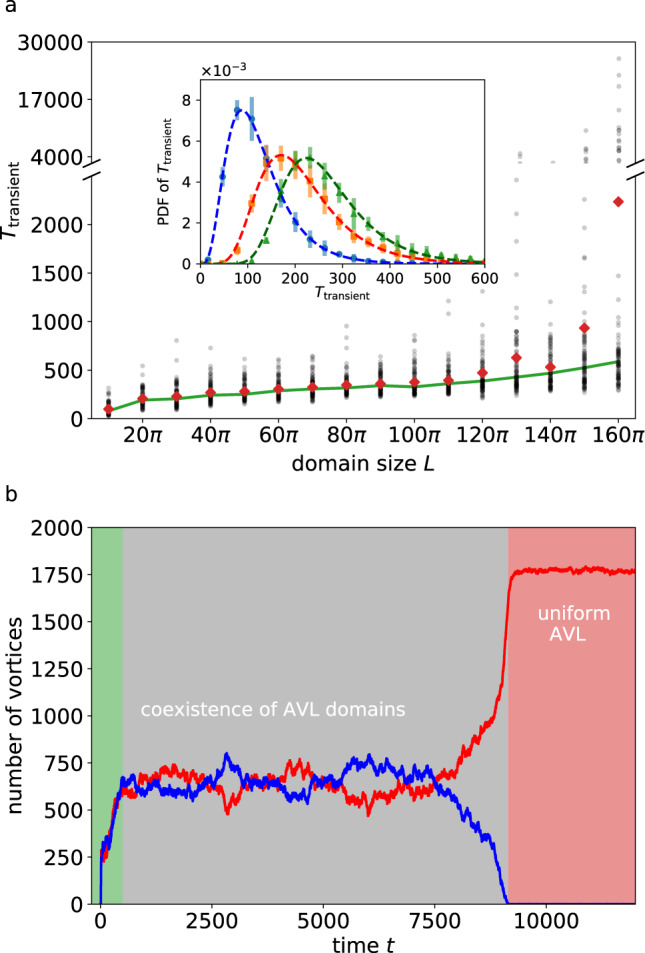
Transient durations of AVL emergence. (**a**) Scatter plot of the transient durations leading to a uniform AVL domain as a function of domain size *L*. The red diamonds and the green curve are the mean and median values, respectively. The change in slope at about *L* = 120*π* marks the domain size where AVL domains of opposite polarity start to become stable. (inset) Probability density functions of the transient durations for *L* = 10*π* (blue circles, *τ* = 54.59, *δ* = 4.97), 20*π* (orange squares, *τ* = 72.78, *δ* = 10.40) and 40*π* (green triangles, *τ* = 72.81, *δ* = 21.67), obtained from 10^4^ simulations each, and the corresponding fits with the theoretically proposed PDF (4) (dashed curves). The error bars correspond to the difference between the maxima and the minima of five subsampled PDFs (Methods). (**b**) The time series of the number of positive (red) and negative vortices (blue) for a simulation with large domain size (*L* = 160*π*). The green, gray, and red regions denote the initial transient, the coexistence of AVL domains of opposite polarity, and the final uniform AVL, respectively.

Next, we characterize the emergence of uniform AVLs as a function of system size. To this end, we determined the transient time until a uniform AVL is formed for an ensemble of 100 simulations for each system size, covering domain sizes between *L* = 10*π* and *L* = 160*π* (Methods). Figure 4a shows the resulting scatter plot, which demonstrates that the lifetime of the transient state depends sensitively on the initial condition and increases considerably with domain size.

For small domains, this is mainly rooted in the fact that the emergence of a uniform AVL occurs after a spontaneous discrete symmetry breaking through a turbulent transient^48^, which renders the transient time a random variable. In fact, the PDF of transition times is well captured by

4 $$P(T)=\frac{\delta }{\tau }{\left(1-{{{{{{{{\rm{e}}}}}}}}}^{-\frac{T}{\tau }}\right)}^{\delta -1}{{{{{{{{\rm{e}}}}}}}}}^{-\frac{T}{\tau }},$$

where *τ* and *δ* depend on the vortex lifetime and domain size, respectively. This expression can be rationalized from the observation that vortex lifetimes in active turbulence have an approximately exponential distribution^47^. A good estimate for the transient time is the time after which the spontaneous symmetry breaking occurs. Its distribution can be obtained from the PDF of the time it takes for one polarity of vortices to decay, which amounts to computing the maximum survival time of a set of like-signed vortices. Assuming statistical independence of the individual decay processes yields the proposed PDF (4). Figure 4 (inset) shows the corresponding fits for the PDFs of the transient durations obtained from 10^4^ simulations for each domain size, demonstrating an excellent agreement. We note that, contrary to the expectation, the parameter *δ* does not increase quadratically with the linear extent of the system. This could be due to the dynamic nature of the vortex lifetimes, which we assumed to be static. With a greater asymmetry in the number of vortices of both signs, the lifetime of the decaying vortices will likely decrease, more effectively reducing the number of vortices that need to decay for the AVL to emerge. This lowers the value of *δ* from the expected quadratic dependence on the linear system extent.

**Fig. 5:**
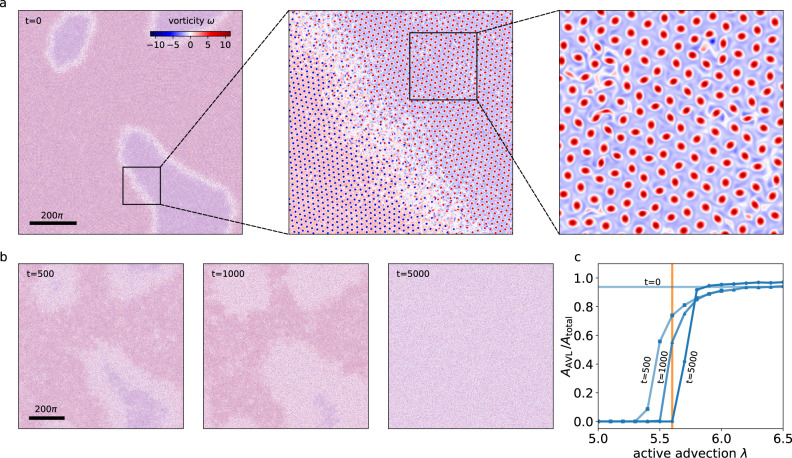
Melting of active vortex lattice domains. (**a**) Transient meta-stable opposite-polarity AVL domains (*λ* = 7, *α* = −0.8, domain size *L* = 1000*π*). The zoom-in shows that these domains are demarcated by an interfacial layer of active turbulence. Panel (**b**) illustrates the melting of the AVL domains shown in panel (**a**) for *λ* = 5.6 after times *t* = 500, *t* = 1000 and *t* = 5000. (**c**) Area fraction of the domains as a function of active advection for times *t* = 500 (squares), *t* = 1000 (triangles) and *t* = 5000 (circles), starting from the configuration (**a**) at time *t* = 0. Note that the transient width of the domain boundaries is controlled by the strength of the active advection. The orange line corresponds to the snapshots shown in panel (**b**).

For sufficiently large domain sizes, an additional effect comes into play: AVL domains with both polarity can coexist for very long times (see, e.g., Fig. 5a and the discussion below). The temporal evolution of AVL clusters is illustrated in Fig. 4b, which shows the number of positive and negative vortices as a function of time (*L* = 160*π*). In this example, two vortex clusters of approximately equal sizes but opposite polarity coexist for more than 8000 nondimensional time units, before a uniform AVL forms. These transient AVL domains explain the extreme outliers in the transient duration which are the cause for the sharp increase of the mean transient time for system sizes beyond *L* = 120*π*.

### Large-scale active vortex lattices

An example of AVL domains emerging from random initial conditions is shown in Fig. 5a for a domain of size *L* = 1000*π* [Supplementary Video (3)]. The slowly evolving lattice domains are separated by a highly dynamic interfacial area of active turbulence, which corresponds to the liquid phase. These highly dynamic domain boundaries play a prominent role in the melting of AVL domains, which can be induced, for example, by decreasing the active advection parameter. As active advection is decreased, the AVL domains melt, and the turbulent domain boundaries spread in area (Fig. 5b).

A natural order parameter to characterize the melting is the fraction of area covered by the AVL domains *A*_AVL_/*A*_total_ (Methods). To illustrate this transition, we evaluate the AVL area fraction as a function of active advection (at a fixed activity *α* = −0.8) for different times, which is shown in Fig. 5c. Below *λ* ≈ 5.6, the AVL domains melt completely into a statistically isotropic liquid phase. Above a critical value of *λ* ≈ 6.0, almost the entire domain is covered by vortex lattices, with the area between the lattices of different polarity occupied by a layer of active turbulence. In between, there is a consistent, but slow decrease in the area of this boundary layer as advection is increased. This evolution of AVL domains is qualitatively similar to coarsening in classical systems^66^.

## Discussion

Using large-scale simulations, we have mapped out the dynamical states of a generalized Toner-Tu model for active fluids. Our analysis establishes the emergence and melting of self-organized active vortex lattices. Depending on the path through the parameter space spanned by the active advection and the activity parameters, we find two distinct transition scenarios. In the first transition scenario, we observe a coexistence of dynamical states, i.e. the active vortex lattice dynamically dissolves into active turbulence and then re-emerges. In the second scenario, we observe a hysteretic transition between AVL and AT states.

On larger domains, we find evidence for solid order within the AVL regime, i.e. we established the existence of active vortex crystals. When changing parameters according to the first scenario discussed above, we observe the unbinding of defect pairs, which goes along with the loss of long-range orientational order before melting into a liquid. This indicates a melting of the AVCs through an intermediate hexatic phase. On very large domains, broken-symmetry AVL domains of opposite polarity emerge, whose melting results from the spreading of the turbulent interfacial layers. Generally, our results reveal the roles of nonlinear advection and activity in the complex self-assembly and melting of AVLs, and highlight connections between phase transitions in active matter and their classical equilibrium counterparts.

Experimental tests of our predictions could be an exciting direction for future work. Stable vortex lattice states in bacterial systems have been experimentally realized^67^ and numerically studied^68^ by means of regular arrays of obstacles on a substrate. There are also experimental systems where a vortex lattices can self-organize without the aid of patterned substrates. For instance, dense suspensions of spermatozoa show both, active turbulence^69^ as well as self-organized regular vortex lattices^9^ and are arguably the best candidates to test our prediction on AVL-AT transitions. Although previous experiments^9^ so far suggest a liquid-like arrangement of vortices, rather than solid-like or hexatic, it may be possible to achieve solid order through a careful tuning of experimental conditions^70^. For instance, the type of sperm cells as well as the intracellular ionic concentrations could affect the nature of sperm motility^71^. Furthermore, the analysis of crystalline order in such systems would also require conducting experiments on large domains. If a vortex crystal phase is achieved, the activity can be tuned, for instance, by changing the motility through the ambient temperature^72^ to induce a potential melting transition. The preferred handedness of sperm cells on planar surfaces^73^ precludes the observation of a spontaneously broken discrete symmetry of the vortices. This could be alleviated by confinement between two walls. Such experiments will significantly enhance our knowledge of crystalline order, not just in active matter, but in out-of-equilibrium systems in general.

## Methods

### Simulation details

We perform direct numerical simulations of the vorticity field *ω* = ∇ × **u** on a periodic domain by using a fully dealiased pseudo-spectral algorithm. The mean velocity $\langle {{{\bf{u}}}}\rangle$ is integrated separately. The corresponding evolution equations follow from (1) and take the form:

5 $${\partial }_{t}\omega +\lambda {{{{{{{\bf{u}}}}}}}}\cdot \nabla \omega =	 \, -\left(\right.1+{{\Delta }}{\left)\right.}^{2}\omega -\alpha \omega -\beta \nabla \times \left({\left|{{{{{{{\bf{u}}}}}}}}\right|}^{2}\ {{{{{{{\bf{u}}}}}}}}\right),\\ {\partial }_{t}\langle {{{{{{{\bf{u}}}}}}}}\rangle =	 \,-\left(\right.1+\alpha \left)\right.\langle {{{{{{{\bf{u}}}}}}}}\rangle -\beta \left\langle {\left|{{{{{{{\bf{u}}}}}}}}\right|}^{2}\ {{{{{{{\bf{u}}}}}}}}\right\rangle .$$

**Fig. 6:**
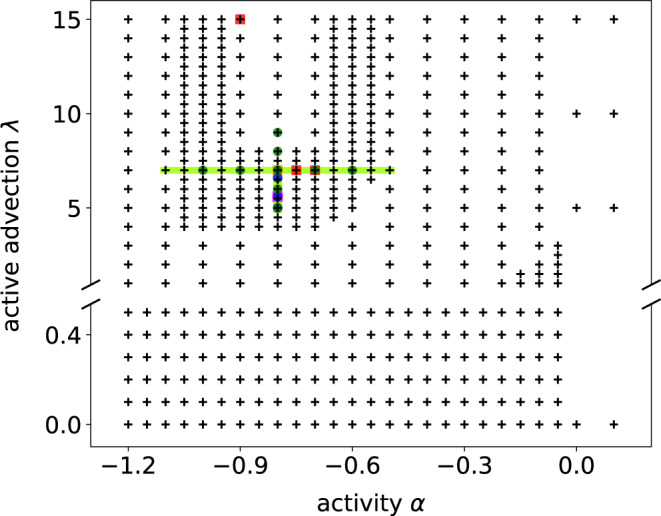
Overview of simulation parameters. The crosses show the parameter configurations used to obtain the phase diagram (Fig. 1, domain size *L* = 20*π*). Each cross represents simulations with two different initial conditions, as noted in the text. The PDFs in Fig. 1f are based on simulations with parameters marked with blue circles (*L* = 20*π*). The phase transition curves Fig. 1g and h are based on simulations with parameters marked with a light green line (*L* = 20*π*). The parameters used in Fig. 2 are indicated by red squares (*L* = 1000*π*) and the parameters used in Fig. 3 by dark green circles (*L* = 200*π*). Figures 4 (*L* = 10*π* − 160*π*) and 5a (*L* = 1000*π*) are based on the parameter choice shown in yellow. Figures 5b (*L* = 1000*π*) and 1e (*L* = 20*π*) are based on the parameter choice indicated by a pink square. See also Table 1 for the parameter values.

We solve (5) with a fourth-order Runge-Kutta method for time stepping combined with an integrating factor for the linear terms. Our code is parallelized using GPUs (graphics processing units) in order to accelerate the computations. For the results discussed in the main text, the parameter values are listed in Table 1 and shown in Fig. 6.

**Table 1:** Simulation parameters: Domain size *L*, active advection parameter *λ*, activity parameter *α*, number of grid points *N*, time step Δ*t*. The parameter *β* is set to 0.01 in all simulations. Values in parentheses represent parameter ranges of simulation series.

Figure	*L*	*λ*	*α*	*N*	Δ*t*
1a	20*π*	[0.0, 15.0]	[−1.20, 0.00]	256^2^	0.005
1b	20*π*	8.0	−0.80	1024^2^	0.001
1c	20*π*	3.0	−0.80	1024^2^	0.001
1d	20*π*	0.1	−0.20	1024^2^	0.001
1e	20*π*	5.6	−0.80	256^2^	0.005
1f	20*π*	5.0, 5.6, 6.6	−0.80	256^2^	0.005
1g	20*π*	[5.0, 7.0]	−0.80	256^2^	0.005
1h	20*π*	7.0	[−1.10, −0.60]	256^2^	0.005
2a/d/e	1000*π*	15.0	−0.90	4096^2^	0.005
2b/d/e	1000*π*	7.0	−0.75	4096^2^	0.005
2c/d/e	1000*π*	7.0	−0.70	4096^2^	0.005
3a	200*π*	7.0	−1.00, −0.90, −0.80, −0.70, −0.60	1024^2^	0.005
3b	200*π*	5.0, 6.0, 7.0, 8.0, 9.0	−0.80	1024^2^	0.005
4a	[10*π*, 160*π*]	7.0	−0.80	256^2^, 512^2^, 1024^2^	0.005
4a inset	10*π*, 20*π*, 40*π*	7.0	−0.80	256^2^	0.005
4b	160*π*	7.0	−0.80	1024^2^	0.005
5a	1000*π*	7.0	−0.80	8192^2^	0.005
5b	1000*π*	5.6	−0.80	8192^2^	0.005
5c	1000*π*	[5.0, 6.5]	−0.80	8192^2^	0.005
7	1000*π*	15.0	−0.90	4096^2^	0.005
8	1000*π*	5.6	−0.80	8192^2^	0.005

### Identification of dynamical states and transitions between them

 Figure 1a is obtained from simulations of 477 different parameter configurations as shown in Fig. 6. For each configuration, we use two different initial conditions: a random initial condition and a vortex lattice.

The different dynamical states shown in Fig. 1a are defined as follows. The square lattice, active turbulence and vortex lattice states show obvious qualitative differences as noted in the main text and are easily distinguished visually. The hysteresis in the marginal stability region is identified as such when the simulations are bistable: the simulations starting with random initial conditions result in an active turbulence state whereas a vortex lattice initial condition remains stable. The simulations are checked for convergence until a total simulation time of *T* = 2000 (4 × 10^5^ time steps). The coexistence region is defined by evaluating the PDF of the energy density. If the PDF has two peaks (see, e.g., Fig. 1f), it is defined as a temporally intermittent pattern.

The transition between active turbulence and vortex lattices in small domains (Fig. 1g and h) is characterized as follows. For the transition curves in both increasing and decreasing directions of parameter values, we conduct simulations in the range 5.0 ≤ *λ* ≤ 7.0 and −1.1 ≤ *α* ≤ −0.6. For *λ* = 5 and *α* = −1.1, we start our simulation from random initial conditions. For the rest of the simulations, the final snapshot of the previous simulation is used as the initial condition. Once a statistically steady state is reached (after about *T* = 5000), we collect data for 10^6^ time steps and evaluate the PDF of the energy density. If the PDF has only one peak, the order parameter *T*_AVL_/*T*_total_ takes the value 0 or 1, depending on the phase. Otherwise, the energy density at the minimum between the two peaks of the PDF, ${E}_{\min }$, is evaluated. The order parameter then takes the value of the probability that the energy density *E* is greater than ${E}_{\min }$. This process is repeated five times, and the mean and the standard deviations are used to construct the transition curves and estimate the uncertainties, which are shown in Fig. 1g and h.

### Defects, dynamic Lindemann parameter and orientational correlation

 Figure 2 is obtained from simulations on a 1000*π* × 1000*π* domain starting from random initial conditions. Since the time required to fully crystallize grows rapidly with domain size, we follow the procedure below to obtain converged simulations.

We initialize a simulation with a random initial condition on a small domain (125*π* × 125*π*) and run it for 10^4^ time units. The simulation is then upscaled to a larger domain (250*π* × 250*π*) by tiling the larger domain with four copies of the converged fields from the smaller domain. A random complex noise (respecting the Hermitian symmetry) is added in Fourier space to ensure that the four sub-domains of the new domain are no longer identical. The amplitude of the noise is chosen as

6 $${A}_{{{{{\mbox{noise}}}}}} \sim k\ \exp \left(-\left(\right.k-4{\left)\right.}^{2}\right)$$

where *k* is the wavenumber. This choice ensures that the perturbations are comparably smaller-scale and do not destroy individual vortex structures.

**Fig. 7:**
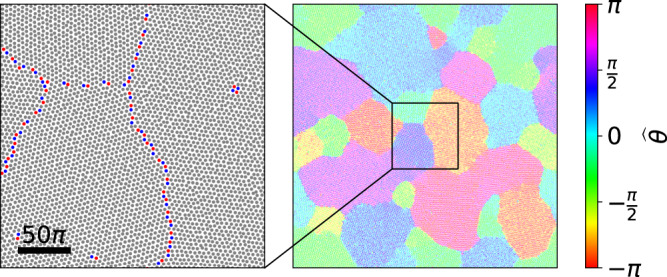
AVL consisting of domains with different lattice orientations (right), obtained from a simulation with *α* = −0.9 and *λ* = 15, following the standard upscaling process described in the Methods. The zoom-in shows the defects between the domains (left).

The upscaling procedure is then repeated until we reach a domain size of 1000*π* × 1000*π*. An exception to this approach is the simulation for the solid phase (Fig. 2a). Here, it is very difficult to obtain a vortex lattice with uniform lattice orientation (Fig. 7). Hence the upscaling process is completed in just one step, where the 1000*π* × 1000*π* domain is constructed from 64 copies of the initial 125*π* × 125*π* domain. The noise is only applied once. The simulations are then evolved until the orientational correlation functions become statistically stationary.

To identify the defects, we first determine the centers of strong vortices^47,74^. By constructing a Voronoi partition (using the Python open source module scipy.spatial.Voronoi) over this vortex core configuration, 5-fold and 7-fold defects are determined^6^.

The orientational order is evaluated from ${\psi }_{i}={\sum }_{j}\exp (6{{{{{{{\rm{i}}}}}}}}{\theta }_{ij})/N(i)$ where *θ*_*ij*_ is the angle between a vortex *i* and its neighbor *j* with respect to an arbitrary axis, and *N*(*i*) is the number of nearest neighbors. Figures 2a, b and c (bottom row) show the angle with respect to the mean orientation:

7 $${\widehat{\theta }}_{i}=\arctan \left(\frac{\Im ({\psi }_{i})}{\Re ({\psi }_{i})}\right)-\left\langle \arctan \left(\frac{\Im ({\psi }_{i})}{\Re ({\psi }_{i})}\right)\right\rangle$$

where *ℑ* and *ℜ* denote imaginary and real parts, respectively, and the mean orientation is determined by an average over all vortices.

The orientational correlation function *G*_6_(*r*) is then evaluated according to (2) in the main text, based on the orientational order. The results are averaged over six snapshots for Fig. 2 and three snapshots for Fig. 3, separated by 1000 time units each. The error bars are obtained by evaluating the standard deviations across the six/three snapshots.

To evaluate the dynamic Lindemann parameter, we obtain 1000 snapshots separated by 0.1 time units after the simulations reached a converged state. For each snapshot, we identify the centers of strong vortices. The trajectory of each vortex core is then tracked for a time period of 50 (solid and hexatic) or 10 (liquid) time units. Only vortices which survive the entire duration of the period are included in the analysis. The dynamic Lindemann parameter is then evaluated following (3) in the main text. To increase statistical convergence, results are additionally averaged over six subsequent time periods, from which we also compute the standard deviation.

Results in Fig. 3 are obtained through simulations on a domain of size 200*π* × 200*π*. Since a complete scan of the parameter space is computationally infeasible for this domain size, two cuts through the solid region are analyzed. Again, each choice of *α* and *λ* is simulated with a random initial condition as well as a vortex lattice initial condition for a total time of *T* = 10000. The orientational correlation *G*_6_(*r*) is calculated according to (2) in the main text.

### Transient durations

To evaluate transient durations (Fig. 4a and b), we conduct simulations starting from random initial conditions until a converged vortex lattice state is reached for each domain size. The convergence is defined as follows. By employing a vortex identification algorithm^47,74^, we obtain a time series of the number of strong vortices of both polarity. A converged vortex lattice is obtained when the number of vortices of either sign reaches 93% of the theoretical maximum number of vortices possible in the domain. To obtain the mean and median transient durations in Fig. 4a, the simulations are repeated 100 times for each domain size and the corresponding mean and median durations are calculated. The PDFs (Fig. 4a inset) are obtained by evaluating the transient durations for three different domain sizes from 10^4^ simulations each, starting from random initial conditions. The error bars correspond to the difference between the maxima and the minima of five subsampled PDFs obtained from 2000 simulations each. The theoretical curves are obtained by fitting (4) to the numerical data. The corresponding values of the free parameters *δ* and *τ* are, respectively, 4.97 and 54.59 for *L* = 10*π* (blue curve), 10.40 and 72.78 for *L* = 20*π* (orange curve) and 21.67 and 72.81 for *L* = 40*π* (green curve).

### Melting transition on large domains

**Fig. 8:**
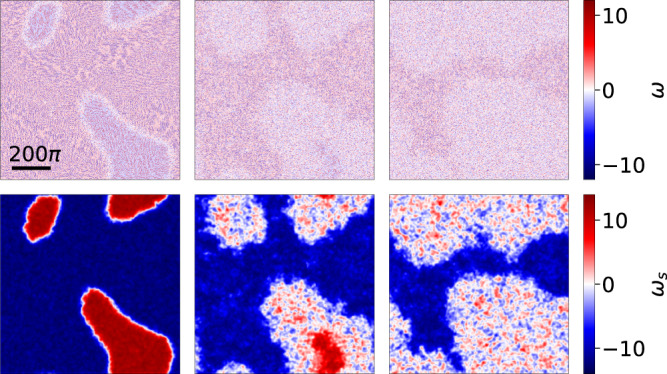
Analysis of melting of AVL domains. Top row shows the vorticity field *ω* for *λ* = 5.6 at different times (initial condition, at *t* = 1000, and at *t* = 2000). Bottom row shows the corresponding smoothed fields *ω*_*s*_.

To evaluate the melting transition curve on large domains (Fig. 5c), we first identify centers of the strong vortices^47,74^. Then an order parameter field is obtained by calculating, for each point (*x*, *y*), the difference between the number of positive and negative vortices within a circle of radius *r* centered at (*x*, *y*) (*r* is about 1.5 times the mean distance between the nearest neighbors). The resulting field is then smoothed using a Gaussian filter with standard deviation *σ* = 12. The original vorticity field *ω* and the smoothed field *ω*_*s*_ are shown in Fig. 8. The turbulent region is then defined as the area where the absolute value of this smoothed field is less than half the maximum value of the field. Once this turbulent region is defined, the order parameter *A*_AVL_/*A*_total_ is calculated by evaluating the fraction of the total area covered by the AVL.

## Supplementary information

Description of Additional Supplementary Files

Supplementary Movie 1

Supplementary Movie 2

Supplementary movie 3

## Data Availability

The data used in this study are available from the corresponding author on request.

## Code availability

The simulation code is available on github (https://github.com/DominikSuchla/2D_active_matter_simulation) under the GNU General Public License v3.0. Post-processing codes that have been used to produce the results of this study are available from the corresponding author on request.
