# Supplementary material for: Emergence and melting of active vortex crystals
Source: Nat Commun. 2021 Sep 24;12:5630. doi: 10.1038/s41467-021-25545-z (PMC8463610; doi:10.1038/s41467-021-25545-z)
Supplement: Supplementary file 1 — Description of Additional Supplementary Files [file 41467_2021_25545_MOESM1_ESM.pdf]

Description of Additional Supplementary Files

File name: Supplementary Video 1
Description: Emergence of an active vortex lattice starting from random initial conditions.

File name: Supplementary Video 2
Description: The active matter system in the marginal stability region between the turbulent and the active vortex lattice states. The right panel shows how the energy density changes with time.

File name: Supplementary Video 3
Description: Evolution of active vortex lattice domains. The inset shows a zoom-in into the turbulent interfacial area.
